# COVID-19 cross-border case and contact tracing activities - experiences and lessons learnt, Germany, April-December 2020

**DOI:** 10.1186/s12889-023-16213-6

**Published:** 2023-07-04

**Authors:** Ida Sperle, Uwe Koppe, Raskit Lachmann, Robert Vonderwolke, Nadine Püschel, Nadine Litzba, Paula Böhm, Janina Stauke, Annika Heck, Jonathan H.J. Baum, Luam Ghebreghiorghis, Gyde Steffen, Ute Rexroth, Maria an der Heiden, Timm Schneider, Inessa Markus

**Affiliations:** 1 Department of Infectious Disease Epidemiology, Robert Koch Institute, Berlin, Germany; 2 Robert Koch Institute, Postgraduate Training for Applied Epidemiology (PAE), Berlin, Germany; 3 ECDC Fellowship Programme, Field Epidemiology path (EPIET), European Centre for Disease Prevention and Control (ECDC), Stockholm, Sweden; 4 Robert Koch Institute, Centre for International Health Protection, Berlin, Germany

**Keywords:** COVID-19, Cross-border contact tracing, Surveillance, Germany

## Abstract

**Background:**

Interruption of transmission chains has been crucial in the COVID-19 response. The Emergency Operations Centre (EOC) at the Robert Koch Institute (RKI) coordinated cross-border case and contact tracing activities at the national level by sharing data with German public health authorities (PHA) and other countries. Data on these activities were not collected in the national surveillance system, and thus were challenging to quantify. Our aim was to describe cross-border COVID-19 case and contact tracing activities including lessons learnt by PHA to adapt the procedures accordingly.

**Methods:**

Case and contact tracing events were recorded using unique identifiers. We collected data on cases, contacts, dates of exposure and/or SARS-CoV-2 positive test results and exposure setting. We performed descriptive analyses of events from 06.04.-31.12.2020. We conducted interviews with PHA to understand experiences and lessons learnt, applying a thematic approach for qualitative analysis.

**Results:**

From 06.04.-31.12.2020 data on 7,527 cross-border COVID-19 case and contact tracing activities were collected. Germany initiated communication 5,200 times, and other countries 2,327 times. Communication from other countries was most frequently initiated by Austria (n = 1,184, 50.9%), Switzerland (n = 338, 14.5%), and the Netherlands (n = 168, 7.2%). Overall, 3,719 events (49.4%) included information on 5,757 cases (median 1, range: 1–42), and 4,114 events (54.7%) included information on 13,737 contacts (median: 1, range: 1–1,872). The setting of exposure was communicated for 2,247 of the events (54.6%), and most frequently included private gatherings (35.2%), flights (24.1%) and work-related meetings (20.3%). The median time delay between exposure date and contact information receipt at RKI was five days. Delay between positive test result and case information receipt was three days. Main challenges identified through five interviews were missing data or delayed accessibility particularly from flights, and lack of clear and easy to use communication channels. More and better trained staff were mentioned as ideas for improving future pandemic response preparedness.

**Conclusion:**

Cross-border case and contact tracing data can supplement routine surveillance but are challenging to measure. We need improved systems for cross-border event management, by improving training and communication channels, that will help strengthen monitoring activities to better guide public health decision-making and secure a good future pandemic response.

## Background

The COVID-19 pandemic put a large strain on public health systems, and required rapid and continuous adaptation of public health measures and mitigation strategies. SARS-CoV-2 is highly contagious and predominantly transmitted by human-to-human transmission, including via long-range airborne transmission [1–4]. Various containment measures were implemented in Germany and elsewhere to interrupt transmission chains, including case finding through testing, isolation of cases and contact tracing. In Germany the responsibility for contact tracing lies locally with the public health authorities (PHA). International communication in infectious disease events relevant to either the International Health Regulations (IHR, 2005) [5] or the Decision 1082/2013/EU [6] is performed by the Robert Koch Institute (RKI). At the RKI, a designated team was established within the COVID-19 Emergency Operations Centre (EOC) to coordinate cross-border communication.

To date, there is little publicly available information on cross-border COVID-19 case and contact tracing. Germany does not collect COVID-19 cross-border case and contact tracing activities in its national surveillance system, thus they are challenging to measure and quantify. In a globalized world, it is however of importance to increase evidence and ensure that countries are prepared for cross-border communication and collaboration in pandemic responses.

In our previously published paper on cross-border contact tracing in Germany (February-April 2020) we found an increase in cross-border COVID-19 contact tracing activities with increasing COVID-19 incidence [7]. Further, results suggested the time delay from exposure to information to the RKI exceeded the median COVID-19 incubation time [7]. Our aim was to expand on these analyses [7] and thereby provide evidence on case and contact tracing activities in Germany during the COVID-19 pandemic between 6 April-31 December 2020 to guide future action. More specifically, our objectives were to:

1. Describe the magnitude and key characteristics of cross-border case and contact tracing activities focusing on: exposure (country and setting), number of contact persons and cases, and communication channels;
2. Analyse the duration of the information transfer during cross-border case and contact tracing and compare it with epidemiological recommendations;
3. Identify challenges and lessons learnt from the perspective of PHA in Germany to understand barriers to efficient cross-border case and contact tracing.

## Methods

### Cross-border case and contact tracing events

COVID-19 case and contact person definitions corresponded to respective definitions used by the country sharing the data. In Germany, during the study period, close contacts were defined as persons who had been in contact with a case during their infectious phase (from two days before symptom onset until 10 days after; for asymptomatic cases the date of sampling was used) [8]. Information on close contacts or cases that was received too late (considering the infectious period and possibility to implement preventive measures) were not transferred. The decision to pause and initiate contact tracing from flights, was agreed upon in the crisis committee. A detailed description of the cross-border communication of case and contact tracing events was described in the previous publication [7]. In short, information was shared through two main communication channels. Between the RKI and countries in the European Union (EU)/European Economic Area (EEA) information was transferred via the Early Warning and Response System (EWRS), a single-window communication platform which allowed secure transfer of personal data. For World Health Organization (WHO) Member States outside EU/EEA the data were transferred using an encrypted exchange server (Cryptshare) via the IHR National Focal Point (NFP) system. Within Germany, data were also transferred using Cryptshare, either directly between RKI and the PHA or via the federal state level offices. For some federal states which border other countries, already established cross-border networks were potentially used without involvement of the RKI.

Data on cross-border case and contact tracing events transferred through the RKI EOC were routinely collected and documented in an Excel spreadsheet for the purpose of communication. The following information was documented: number of contacts and/or cases, date of contact and/or positive test result, exposure context, type and date of communication, and involved stakeholders.

### Quantitative methods

#### Data extraction

We extracted information from the Excel spreadsheet used in the EOC on all cross-border case and contact tracing events from 6 April-31 December 2020 using a comprehensive standardised data extraction form excluding events not specifying the number of contacts or cases.

#### Data analysis

We performed a descriptive analysis of cross-border case and contact tracing events in STATA™ (software version 17.0, StataCorp) focusing on the following outcomes:

- Number of case and contact tracing events;
- Number of contacts and cases;
- Exposure context and country;
- Communication channel used;
- Time delay between date of exposure (or date of positive test for cases) and date of communication from RKI to the responsible PHA in Germany or foreign health authorities.

If the country of exposure was not explicitly reported, the country which initiated the communication was recorded. For all events for which exposure was transport (e.g. international flights and ships), the country of departure was recorded as country of exposure. If the cross-border case and contact tracing event included more than one person with different dates for the exposure or positive test results, the latest date was used. To contextualize the data, we used different phases as defined in previous publications [9, 10]:

- Phase 1: March-May 2020 (calendar weeks 5–20 2020, spring).
- Phase 2: June-September 2020 (calendar weeks 21–39 2020, summer).
- Phase 3: October -December 2020 (calendar weeks 40 and onwards, autumn/winter).

### Qualitative methods

#### Data collection

Information on experiences on cross-border case and contact tracing events was collected through interviews with a purposive sample of respondents from the PHA. Participants were selected based on their knowledge and experiences with the topic. The 16 federal states in Germany were asked by the RKI to inform their local PHA about the study and the possibility to participate on a voluntary basis. Phone interviews were conducted by one interviewer over a five-month period from September 2021-January 2022. A semi-structured interview guide was used mainly covering challenges and lessons learnt in the areas of communication, work load, collaboration with RKI and at the local level. All interviews were audio recorded with participant’s consent, and followingly transcribed by one person. Personal information and the anonymised recordings and transcriptions were saved on a separate secured drive. Recordings were deleted after transcription.

#### Data analysis

Thematic analysis as outlined by Braun and Clarke (2006) was used to identify response patterns in the interviews to be developed into recurrent themes [11, 12]. The theoretical position applied for the analysis was the realist method, where experiences, meaning and realities of the participants are reported [11, 12].

#### Ethical approval and data protection

Data on cross-border case and contact tracing events were collected within the legal framework of the German Infection Protection Act (IfSG), the EU Decision 1082/2013 and the IHR (2005). The qualitative data collection was approved by the data protection officer at the RKI.

## Results

### Cross-border case and contact tracing events

We analysed 7,527 cross-border case and contact tracing events from 6 April to 31 December 2020 in Germany. Germany initiated communication 5,200 times, and other countries 2,327 times (Table 1). Communication from other countries was most frequently initiated by the neighbor countries Austria (n = 1,184, 50.9%), Switzerland (n = 338, 14.5%), and the Netherlands (n = 168, 7.2%). In total, 3,719 events (49.4%) included information on 5,757 COVID-19 cases (median 1, range: 1–42). In 3,027 events (81.4%) one case was communicated, and in 692 events (18.6%) two or more cases were communicated. Among all recorded 7,527 events, 65.1% (n = 4,902) included communication of both cases and contacts.

**Fig. 1:**
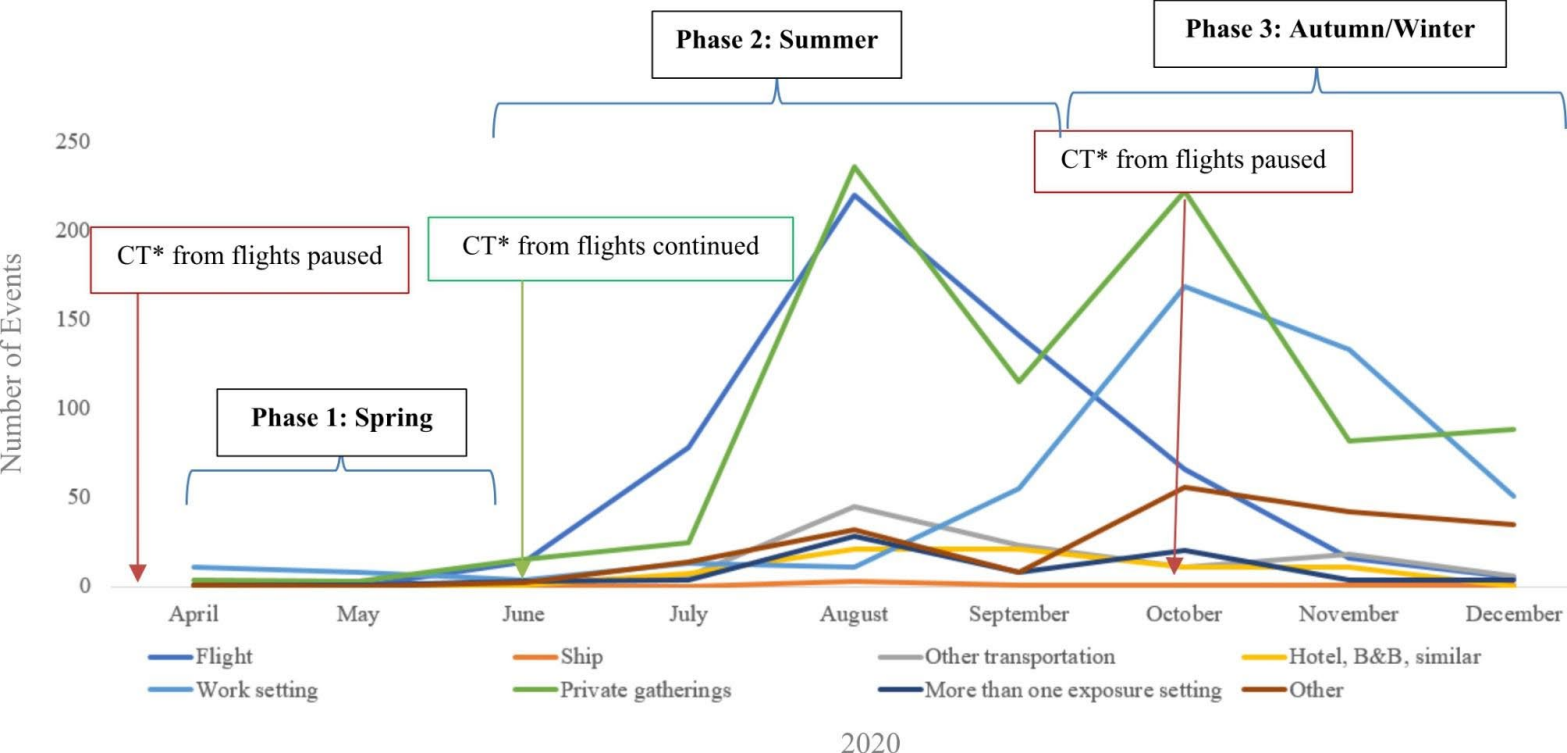
Exposure settings in cross-border contact tracing over time (6 April-31 December 2020), n = 2,247 *contact tracing

Overall, 4,114 events (54.7%) included information on 13,737 contacts (median: 1, range: 1–1,872). The event which involved 1,872 contact persons was a cruise ship with around 2,900 persons from different countries. Excluding the event with the cruise ship, there was a total of 4,113 events with 11,865 contacts (median:1, range: 1-450). In 1,927 events (46.8%) two or more close contacts were communicated (median: 3, range: 2 − 1,872). For more than half of the events which included information on contact persons (57.9%) the country of exposure was outside Germany (Table 1), of which the most frequently reported were Austria (n = 783), Switzerland (n = 201), Kosovo (n = 190), and Croatia (n = 128). The setting of exposure was communicated for 2,247 of the events (54.6%). The most common exposure contexts were private gatherings (35.2%), followed by flights (24.1%) and work-related meetings (20.3%) (Table 2), but this varied over time. Flights as exposure increased in frequency over the summer period (July-September 2020), but contact tracing from flights was paused from April-June and in October again until end 2020. Private gatherings were reported as a frequent exposure setting throughout 2020 (Fig. 1).

As illustrated in Fig. 1 the numbers and exposure settings varied over time. The phase one of the COVID-19 pandemic (March-May 2020) was characterised by an increasing number of COVID-19 cases in Germany [9]. To be able to focus the PHA resources on responding to the pandemic on the ground contact tracing from flights was paused during phase one. Phase two (June-September 2020) of the pandemic was characterised by a low number of COVID-19 cases in Germany. This was also reflected in the cross-border case and contact tracing data for most categories except for exposure during private gatherings and during flights. The decrease in COVID-19 cases during phase two allowed the continuation of contact tracing from flights, until it was paused again in October 2020. During the third phase of the pandemic (October-December 2020) an increase in COVID-19 cases occurred, and was also followed by a lockdown, leading to drop in events with flight as exposure and also private gatherings (Fig. 1).

The time delay between the date of exposure and the date on which the RKI forwarded the received information of contacts to PHA or foreign health authorities was five days (median) [interquartile range (IQR) 4;8]. For COVID-19 cases, the time from positive test to the forwarding of information by the RKI was three days (median) [IQR: 2;4].

**Table 1:** Number of events, contact persons and cases and days of time delay by country of initial communication and country of exposure, 6 April to 31 December 2020

		Number of events n (%)	Contact persons n (median [IQR])	Cases n (median [IQR])	Days of time delay median [IQR] (contacts)	Days of time delay median [IQR] (Cases)
	**Total**	7,527 (100)	13,737 (1[1;3])	5,757 (1[1;1])	5 [4;8]	3 [2;4]
**Country of initial communication of cases/contacts** **(n = 7,527)**	**Germany**	5,200 (69.1)	9,821 (1[1;3])	4,039 (1[1;1])	6 [4;8]	3 [2;5]
	**Abroad**	2,327 (30.9)	3,916 (1[1;2])	1,718 (1[1;1])	5 [3;7]	2 [1;4]
**Country of exposure (n = 4,114)**	**Germany**	1,668 (40.5)	7,660 (1[1;2])	3,267 (1[1;1])	6 [4;8]	3 [1;4]
	**Abroad**	2,382 (57.9)	5,882 (1[1;3])	2,417 (1[1;1])	5 [4;8]	3 [2;5]

**Table 2:** Cross-border contact tracing events, number of communications with health authorities, and contact persons by exposure setting, 6 April to 31 December 2020, n = 7,527

	Total number of events n (%)	Authorities the RKI was in contact with and communication channel used (n)					Number of contact persons (n)
			German PHA (Cryptshare)	EU/EEA (EWRS)	IHR NFP (Cryptshare)		
**Total**	7,527 (100%)		6,436	6,565	1,472		13,737
**Exposure setting**							
Flights*	967 (12.9%)		985	831	348		1,913
**Ships**	14 (0.2%)		16	30	26		1,926
**Other means of transportation**	164 (2.2%)		163	170	38		564
**Hotel, B&B, or similar**	183 (3.4%)		227	196	12		731
**Work related meetings/work commute**	607 (8.1%)		567	613	51		1,149
**Private visits/gatherings**	884 (11.7%)		782	629	292		2,749
**Several exposures**	141 (1.9%)		140	141	20		351
Other**	235 (3.1%)		175	206	37		351
**Unknown/not reported**	4,332 (57,6%)		--	--	--		--

*In Germany, contact tracing after exposure in flights was paused from 18 March − 14 June 2020 and from 20 October 2020 to 11 February 2021, ** Some of the most frequently reported settings included hospitals and school settings

### Challenges and lessons learnt – perspectives from local PHA

Five participants from PHA were interviewed. The level of experience varied but all had been involved with cross-border COVID-19 case and contact tracing. Three key themes were identified: data quality, training in the area of preparedness for pandemic response and communication.

#### Data quality

The respondents expressed overall satisfaction with the quality of the data provided to them. However, some reported issues concerning missing accessibility data such as phone numbers or email addresses. This meant that timely contact tracing was not possible, as expressed by one participant:

*“…then there is also nothing we can do when we don’t have a phone number. We definitely need a phone number or an email address. Writing to a postal address will take minimum four days, that does not make sense.”* (participant 4).

A few of the participants raised concerns about timeliness. Data was needed for action and after a certain delay, contact tracing activities were no longer considered useful. This was in particular stressed for contact tracing after exposure on flights, for which an additional challenge was provision of timely and complete data from airlines. Sometimes no information could be retrieved from airlines which was a cause of frustration.

*“The biggest problem we had was with the airlines. It was difficult to get the necessary lists, we had to follow up again and again. That was a lot of work and didn’t work well. It was very frustrating and difficult. It would be nice if this could be improved.“* (participant 5).

#### Communication

Two participants underlined the importance of established channels of communication with other countries.

Three participants mentioned the potential additional delay caused by information flowing through multiple stakeholders, e.g. PHA, federal state level and RKI. The participants questioned whether this is needed, or if COVID-19 warrants an exception:

*“…but the federal state level office is our middle partner and is of course interested in contact tracing [.] but somehow it does not make sense that the RKI forwards information to them and then us. […] There are too many steps in between, but the federal state level office of course sees this differently.”* (participant 4).

Regarding communication channel, two participants mentioned that after familiarising themselves with Cryptshare it worked well. But three expressed unhappiness with the secure data server. As expressed by one participant:

*“.yes Cryptshare, I think that not even half of the emails are read, I can imagine that. So, when RKI just sends something per Cryptshare to a PHA, I bet that half of the PHA do not even open it, or even more, I would guess three quarters.”* (participant 4).

This was echoed by another participant who found the server unintuitive and time consuming. An online platform or portal with pre-defined fields for insertion of information necessary for efficient cross-border case and contact tracing would be better.

Lack of recommendations after exposure in busses was pointed out, and guidance similar to that for contact tracing for flights would be very welcome.

#### Training and preparedness for pandemic response

Three of five participants expressed having gone through a “learning by doing” process. Due to the high workload and intensity of the pandemic, there was little time for training. One participant explained that training new colleagues was labour intensive, as people hired often lacked experience. In addition to the information available from the RKI website, the participant explained that ready-to-use training materials would be helpful as they would also contribute to a standardisation:

*“…This would be very helpful and would help reduce the workload for all PHA at once, to have a staple of up-to-date documents or slides for training – with basic information about contact tracing, the virus and incubation time […] this would probably be better than coming up with everything individually for the training”* (participant 5).

Other ideas mentioned for being better prepared for a future pandemic response were the RKI containment scouts [13], who were hired via RKI in spring 2020 to assist PHA with case and contact tracing activities:

*“I think the RKI containment scouts were really good, and I think that RKI should maintain these and train people who are responsible for this. This is something I would really welcome, meaning that for every federal state there is one pandemic scout who is trained to support the PHA and who can then train the rest of the people through already developed materials. I think that would be really great.”* (participant 2).

## Discussion

In this study we described 7,527 cross-border case and contact tracing events due to the COVID-19 response in Germany from April-December 2020. A total of 5,757 cases and 13,737 contacts were communicated, with private gatherings being the most frequently reported setting of exposure, followed by flights and work-related meetings. This fluctuated slightly over time, reflecting implemented changes in the EOC such as pausing contact tracing from flights and possibly the COVID-19 pandemic phases [9, 10].

The increase in cases in Germany during the first and third phases of the pandemic, led to a pause in contact tracing after exposure during flights in order to focus public health measures on the ground. The third phase was characterised by measures including physical distancing and measures to reduce travel. These developments may have contributed to the drop in events associated with flights and private gatherings as exposure.

The most frequent setting of exposure from 6 April- 31 December 2020 was private gatherings while these were only the third most frequently reported exposure setting from 3 February-5 April 2020 [7]. Other studies have confirmed SARS-CoV-2 exposure in households pose a high-risk for transmission [14, 15]. While cross-border contacts are mostly not (permanent) household contacts, contact in private settings can be comparable to that of household contacts. These events could be prioritised in situations with limited capacity for cross-border case and contact tracing activities.

We found that the median delay from exposure to forwarding of information by RKI equals the average incubation time for COVID-19 (3–6 days) [16, 17]. However, additional delay is inevitable as it takes time to process information for the recipient (either PHA or foreign health authorities). Ideally the communication of information on COVID-19 cases and contacts needs to be more timely to efficiently break transmission chains. Compared to the findings in the previous publication [7] the median number of days delayed decreased from eight days (during 3 February to 5 April 2020) to five days (6 April to 31 December 2020) for contacts. This might reflect that PHA and other countries were becoming more familiar with the cross-border case and contact tracing processes. While the data may reflect some improvement, there remained issues with communication and delay, in particular with flight exposure due to the known issue of inadequate standardised passenger data collection [18]. This was expressed as a large source of frustration among the interview participants, and time and workload required for contact tracing from flights is an issue already reported from data early in 2020 [7]. The process and coordination around contact tracing from flights need to be improved to make it more efficient in the future.

The RKI containment scouts initiative [13] was mentioned as an important and helpful resource during the pandemic response. Securing sufficient (well-trained) staff for cross-border case and contact tracing was a challenge reported by the PHA. A standardised training for all PHA, for example in the form of up-to-date slides with basic information on contact tracing would be helpful in reducing workload and improving training of staff in PHA. The work intensity with cross-border case and contact tracing activities ultimately triggers the question about digital solutions for contact tracing. There are plans on EU level to establish and facilitate cross-border contact tracing that hold promise for the future [19]. This would provide important support in terms of interrupting chains of transmission, also cross-border, and should be in line and support the official reporting channels as outlined in the German Infection Protection Act (Infektionsschutzgesetz; IfSG) and the International Health Regulations (IHR).The national surveillance system in Germany focuses on events in the country, but does not quantitively cover the cross-border aspect. Experiences and lessons learnt from the pandemic confirm the importance of the cross-border activities, but actual public health impact remains unknown. In order to make the best use of these data, a more suitable database from which the data could be drawn and included in the national surveillance would be needed. This database should include pre-defined fields for the necessary information for efficient case and contact tracing activities including date and setting of exposure (or date of positive test and/or symptom onset), number of cases and contacts, and number of countries involved. Internationally agreed standards for reporting would allow comparison across countries and over time. While the EWRS platform is a good example of a messaging system for safe communication of personal data, it is only used in the EU/EEA countries, and communication via WHO IHR and in Germany remains cumbersome.

### Limitations

Our data are subject to limitations. Most importantly, only contacts and cases that are communicated via the RKI were included in our analysis, and the number of events is likely to be higher than reported here.

Important information such as exposure setting and country was frequently not communicated. To compensate for the latter, the country initiating the communication was regarded as country of exposure. However, this may often not be the case and was an important limitation for our analysis. The time span between forwarding the information and action on the receiving end was unknown. If data were received too late in order to initiate public health measures the event was excluded from the analysis. This means that the time delays are underestimated by definition.

The COVID-19 pandemic is changing rapidly, and we were only able to analyse data from 2020. Many significant changes have occurred throughout 2021 and 2022 with new variants of concern emerging such as B.1.617.2 (Delta Variant) (December 2020) and B.1.1.529 (Omicron Variant) (November 2021), and importantly the roll-out of COVID-19 vaccination (in Germany from end of 2020). However, the trend of cross-border case and contact tracing events as well as the impact of associated challenges are most likely transferrable to 2021 and 2022.

Due to the high workload in the PHA we were only able to recruit five interview participants. While repeated response patterns were identified across interviews, which is indicative of saturation, more interviews could potentially have improved internal validity of the qualitative results reported. The fact that a scientist from RKI conducted the interviews may have resulted in social likeability bias and fostered more positive feedback than might have been expressed if the interviews had been done by someone outside RKI.

## Conclusions

The analysis of cross-border case and contact tracing events underlines the importance and complexity of cross-border case and contact tracing for the pandemic response in Germany. The activities are not part of the routine surveillance system for COVID-19, and are challenging to measure. Improved systems for cross-border events management, both in terms of communication but also monitoring and quantification is needed to improve and guide public health decision-making and secure a good future pandemic response.

## Data Availability

The datasets used and/or analysed during the current study available from the corresponding author on reasonable request.
